# 

**Published:** 1895-01

**Authors:** 


### TABLE OF CONTENTS.

ORIGINAL COMMUNICATIONS. PAGE

History of the Discovery of Modern Anæsthesia. By DR. THOMAS FILLEBROWN, Boston . . . 1

The Relative Penetrating Power of Coagulants. By JAMES TRUMAN, Philadelphia . . . 11

An Address upon the Fiftieth Memorial Celebration of the Discovery of Anæsthesia. By DR. JAMES E. GARRETSON, Philadelphia . . . 20

The Physiology of Hypnotism. (*Continued.*) By ALFRED H. PORTER, D.D.S., Philadelphia . . . 27

A Method of filling Root-Canals. By DR. JAMES H. DALY, Boston . . . 31

REPORTS OF SOCIETY MEETINGS.

American Academy of Dental Science . . . 33

Academy of Stomatology . . . 39

Memorial Celebration of the Fiftieth Anniversary of the Discovery of Anæsthesia . . . 50

EDITORIAL.

The Fiftieth Memorial Celebration . . . 58

NOTES AND COMMENTS . . . 61

CURRENT NEWS.

Connecticut Valley Dental Society . . . 63

Woman's Dental Association of the United States . . . 63

Recent Patents . . . 64

